# Metagenomics Approaches for the Detection and Surveillance of Emerging and Recurrent Plant Pathogens

**DOI:** 10.3390/microorganisms9010188

**Published:** 2021-01-16

**Authors:** Edoardo Piombo, Ahmed Abdelfattah, Samir Droby, Michael Wisniewski, Davide Spadaro, Leonardo Schena

**Affiliations:** 1 Department of Agricultural, Forest and Food Sciences (DISAFA), University of Torino, 10095 Grugliasco, Italy; edoardo.piombo@gmail.com; 2 Department of Forest Mycology and Plant Pathology, Uppsala Biocenter, Swedish University of Agricultural Sciences, P.O. Box 7026, 75007 Uppsala, Sweden; 3 Institute of Environmental Biotechnology, Graz University of Technology, Petersgasse 12, Graz 8010, Austria; ahmed.abdelfattah@tugraz.at; 4 Department of Ecology, Environment and Plant Sciences, University of Stockholm, Svante Arrhenius väg 20A, Stockholm 11418, Sweden; 5 Department of Postharvest Science, Agricultural Research Organization (ARO), The Volcani Center, Rishon LeZion 7505101, Israel; samird@volcani.agri.gov.il; 6 U.S. Department of Agriculture—Agricultural Research Service (USDA-ARS), Kearneysville, WV 25430, USA; wvwisniewski@gmail.com; 7 Department of Biological Sciences, Virginia Technical University, Blacksburg, VA 24061, USA; 8 AGROINNOVA—Centre of Competence for the Innovation in the Agroenvironmental Sector, University of Torino, 10095 Grugliasco, Italy; 9 Department of Agriculture, Università Mediterranea, 89122 Reggio Calabria, Italy; lschena@unirc.it

**Keywords:** surveillance, plant pathogens, metabarcoding, metagenomics, detection

## Abstract

Globalization has a dramatic effect on the trade and movement of seeds, fruits and vegetables, with a corresponding increase in economic losses caused by the introduction of transboundary plant pathogens. Current diagnostic techniques provide a useful and precise tool to enact surveillance protocols regarding specific organisms, but this approach is strictly targeted, while metabarcoding and shotgun metagenomics could be used to simultaneously detect all known pathogens and potentially new ones. This review aims to present the current status of high-throughput sequencing (HTS) diagnostics of fungal and bacterial plant pathogens, discuss the challenges that need to be addressed, and provide direction for the development of methods for the detection of a restricted number of related taxa (specific surveillance) or all of the microorganisms present in a sample (general surveillance). HTS techniques, particularly metabarcoding, could be useful for the surveillance of soilborne, seedborne and airborne pathogens, as well as for identifying new pathogens and determining the origin of outbreaks. Metabarcoding and shotgun metagenomics still suffer from low precision, but this issue can be limited by carefully choosing primers and bioinformatic algorithms. Advances in bioinformatics will greatly accelerate the use of metagenomics to address critical aspects related to the detection and surveillance of plant pathogens in plant material and foodstuffs.

## 1. Plant Disease Diagnosis

### 1.1. A Growing Global Problem

The trade and movement of plant material (seeds, fruits, and vegetables) provide opportunities for plant pathogens to transcend international boundaries and spread to new areas. Worldwide, the number of non-native species in different regions is correlated with the volume of imports. In particular, as highlighted in the EUROPHYT (European Union Notification System for Plant Health Interceptions) annual reports, fruit and vegetables are a key source from which plant pathogens are regularly intercepted [1,2]. 

Transboundary plant pathogens can have severe economic consequences on local plant production and negatively impact global food security. It has been estimated that one-quarter of potential global production is lost due to both pre- and postharvest pathogens [3]. These losses directly or indirectly have a significant impact on food security, quality and safety, as well as on food supply chains as a whole [4]. 

Feeding the global population, which is projected to reach 9 billion by 2050 [5,6,7,8], will require a concentrated effort to keep food losses at a minimum. The need to control crop losses has become more pronounced due to challenges imposed by climate change that are affecting plant health by modifying interactions between host plants, pathogens, and the environment [9]. A study conducted in the US to compare future scenarios of food production for 2060–99, clearly indicated decreases in crop yields [10]. The progressive increase in the adoption of sustainable farming practices and the concomitant reduction in agricultural chemical inputs, including pesticides, may also complicate disease management. 

As suggested by the European Regulation 2016/2031 [11], protective measures against pests and diseases of plants should be based on proportionate and effective responses to plant health threats through a combination of technological, ecological and institutional management strategies. Currently, there is a need to establish system-based approaches and tools for early detection, prevention, eradication, control, and management of emerging plant pathogens and to demonstrate their effectiveness and utility. 

### 1.2. Current Challenges

Increasing globalization has made it very difficult to promptly and efficiently detect and prevent new, invasive plant disease outbreaks, especially when it comes to certain types of organism. For instance, monitoring and surveillance of seedborne pathogens is particularly difficult. Seeds are generally shipped over long distances from a small number of production areas, and this can lead to seedborne diseases spreading quickly from nation to nation [12,13,14]. Similar pathways can be followed by microorganisms associated to plant propagative material, particularly in the case of ornamental plants, which are commonly traded on a global scale. Monitoring of airborne plant pathogens, such as *Fusarium graminearum, Venturia inaequalis, Plasmopara viticola*, and rusts is also difficult since their spores can be easily diffused through long-distance dispersal [15,16]. It has been estimated that 4–11% of the components of fine airborne mass is composed of fungal spores [17]. A further difficulty is posed by the fact that an increasing number of disease outbreaks are caused by previously unknown microorganisms which are identified only after they have caused severe damage in a non-native environment. Examples include Sudden Oak Death (SOD) caused by *Phytophthora ramorum* [18] and Olive Quick Decline Syndrome (OQDS) caused by a specific strain of *Xylella fastidiosa* subsp. *pauca* [19]. Frequently, the center of origin is unknown as in the case of *P. ramorum*, although it has been hypothesized that this pathogen may have originated in remote regions of Asia [20]. Several of the reported emerging pathogens do not cause noticeable damage in their native ecosystems having adapted to and co-evolved with their hosts, and so they are less likely to be detected. For example, isolates of *X. fastidiosa* have been obtained from different plant hosts without causing noticeable disease symptoms [21,22,23]. Moreover, some organisms can be harmless endophytes in their natural habitat, but cause disease when they inhabit new host species of plants. For example, some rhododendron endophytes can induce disease in red oak, flowering dogwood, and lupine [24].

Therefore, it is readily apparent that reliable diagnostic methods are needed to identify risks to plant health so that appropriate plant protection strategies can be developed and administered. Rapid disease diagnosis methods are essential to certify exported goods, inspect imported products, perform disease surveillance and monitoring, and implement containment programs for pathogens (IPPC, 2016). In this regard, metagenomic sequencing has great potential for identifying and monitoring multiple, if not all, potential plant pathogens in a single analysis [25]. The use of metagenomic sequencing, however, is still rare due to several technical problems and challenges that need to be addressed to enable the large-scale implementation of metabarcoding and/or shotgun metagenomics in the diagnosis of bacterial and fungal plant pathogens. Recently, an excellent review containing practical technical guidelines for the implementation of high-throughput sequencing (HTS) in the diagnostics of plant pathogens has been published [26]. In the present review, we aim to focus on practical applications by presenting an overview of the status of HTS-based diagnostics of fungal and bacterial plant pathogens, discuss the challenges that need to be addressed, and provide direction for the future researches. We distinguished methods for the detection of a few related taxa (specific surveillance) or the comprehensive analysis of all of the microorganisms present in a sample (general surveillance). Furthermore, we highlighted the applications that would mostly benefit from the implementation of efficient HTS-based diagnostics.

### 1.3. Current Diagnostic Techniques

There are a number of diagnostic methods that are currently being used to address the need for screening, monitoring, and identification of microorganisms. Disease diagnosis in some host–pathogen interactions can be easily accomplished by symptom observations by trained personnel. In most of cases, however, symptoms are not specific and more accurate identification methods are required. The detection of plant pathogens can be based on the use of moist chambers (high-humidity environments) that promote the growth and sporulation of pathogens (fungi and oomycetes) and their isolation on selective culturing media. Isolation, however, is restricted to facultative parasites and may be very complex in root, soil, and water samples, since fast growing or saprophytic organisms can outgrow and conceal the presence of a primary pathogen [27]. Baiting is a common technique used for the detection of oomycetes, such as *Pythium* and *Phytophthora* species, possessing motile zoospores [28]. After isolation into pure culture, identification is pursued based on colony shape and color, cell morphology, and growth characteristics, which all provide information about the pathogen. Although very different, all traditional methods share a number of drawbacks in that they are labor-intensive, slow, and greatly dependent on the expertise of the analyst. Fo these reasons, microbial identification based solely on microbiological and/or microscopy-based techniques can give inconclusive results. Therefore, molecular identification is often combined with traditional approaches, especially when initial attempts at identification are inconclusive. This typically involves the amplification and sequencing of one or several genes whose sequences are diagnostic for a specific taxa (DNA barcoding regions). 

Polymerase chain reaction (PCR)-based techniques can be utilized for all microorganisms, including unculturable taxa, and are fast, sensitive, and specific [29]. A further improvement in terms of sensitivity, specificity, and reliability has been achieved with real-time quantitative PCR (qPCR) that in addition to providing sequence information also provides quantitative data [30,31,32,33]. Another novel approach is the use of loop-mediated isothermal amplification (LAMP) [34]. Although designing and validating LAMP primers is laborious, LAMP does not require expensive equipment and it is fast and highly specific even when low amounts of pathogen DNA are present in a sample. Notably, when combined with on-site DNA extraction methods, LAMP can be used for molecular detection of plant pathogens directly in the field [35,36,37,38,39]. Recently developed nanotechnologies have also provided a new method of detecting nucleic acid sequences [40,41]. For example, Yao et al. [42] used fluorescent silica nanoparticles combined with antibodies to determine the presence of *Xanthomonas axonopodis* pv. *vesicatoria*, which causes bacterial spot disease in Solanaceae plants. The diagnosis of some diseases can also be accomplished by detecting specific miRNAs associated with plant response to a pathogen, using the small non-coding transcripts as reliable biomarkers of infection [43,44].

Even if molecular diagnosis methods are currently the most sensitive means of detecting and identifying pathogens, only one or few target pathogens can be detected in a single reaction mixture [45]. Thus, molecular methods are often referred to as “targeted methods”. This category of detection methods also includes micro- and macroarray-based approaches. These latter approaches can simultaneously detect a large number of microorganisms, but have never been widely applied due to their complexity and difficulty in interpreting their results [46]. Furthermore, as with other molecular techniques, they are based on the use of target-specific primers and/or probes and so can only be used to detect well characterized microorganisms and are not designed to identify and study new species still unknown to science.

In contrast to targeted methods, metagenomic-based approaches have the ability to detect the presence of virtually all microorganisms in a single analysis, and thus represent a truly untargeted method. This makes metagenomic-based approaches very suitable for identifying the presence of quarantine pathogens that may be present in plant tissues, seeds, and food products.

## 2. High-Throughput Sequencing (HTS)

### 2.1. Applications

HTS has the potential to be efficiently used for the surveillance and detection of plant pathogens. It can be used to identify potentially harmful microorganisms, indicating the need for more specific analyses, while also providing comprehensive data on the microorganisms that are naturally present in an agricultural or environmental sample. This knowledge can be used to differentiate between opportunistic or non-harmful microorganisms and pathogens and is also useful for tracking disease outbreaks.

Researchers have been using HTS metabarcoding and shotgun metagenomics for a variety of purposes for more than a decade and several reviews on the subject are available [47,48,49,50]. A few concerns are particularly critical, however, when HTS is used for the detection of pathogens, which is strongly dependent on the objectives of the analysis. Metabarcoding and shotgun metagenomics are the main approaches based on HTS to perform microbial diagnosis and surveillance [26,51]. Metabarcoding is the sequencing of a representative barcode region of a group of organisms in the sample (e.g.: 16S for bacteria and ITS regions for fungi) using universal primer sets. Shotgun metagenomics is the untargeted sequencing of fragmented DNA representing all of the DNA present in a sample.

### 2.2. Sample Preparation

Contamination is a major source of concern when performing DNA extractions for metagenomics analyses, and reviews describing correct sample preparation have been published [46,48]. When the purpose of the analysis is the detection of pathogens, it is important to separate the working areas designated for DNA extraction, PCR and post-PCR, and to properly clean laboratory spaces and equipment with ultraviolet (UV) light and/or DNAse-containing solutions. The evaluation of different extraction protocols on fecal samples in the Human Microbiome Project (HMP) demonstrated that the extraction method used could affect the community composition, probably due to the differential effects of the extraction reagents on cell lysis, which in turn is influenced by the cell wall composition of the microorganisms [52]. Therefore, a preliminary evaluation of extraction methods should be conducted, if possible, prior to an extensive analysis of samples to determine the optimal extraction methods that should be used to obtain an accurate representation of community composition. 

Sequencing of negative controls is another important aspect that should be included in general microbial surveys using a non-targeted approach. In this regard, many commonly used kits have been reported to be contaminated with several genera of microorganisms [53]. This problem can be addressed by the extraction and use of high concentrations of target DNA and the utilization of mock communities as a control [54], even if certified DNA-free extraction kits should always be used. DNA extractions should be performed under a biological hood to avoid contamination from the lab environment. Finally, determining the presence of a select number of microorganisms (targeted approach) significantly reduces concerns about contamination but does not eliminate the need for proper controls. 

### 2.3. Metabarcoding

Given its lower cost, metabarcoding is currently the most popular method to study fungal and bacterial populations and it is likely to become the most applied technique in diagnostic metagenomics. Metabarcoding data require less storage space than shotgun metagenomics, as well as less computational power, and it is easier to deal with, from a bioinformatic point of view. Furthermore, metabarcoding has the potential to detect and identify virtually all microorganisms, including rare and low abundance taxa, and its efficiency may be further increased by hybridization-based capture of target sequences [55]. Moreover, by spiking known quantities of control DNA to samples before the DNA extraction, it is possible to quantify detected organisms [56], even if variability in amplicon length, GC content, DNA secondary structure and extraction protocol can still introduce biases and greatly reduce the accuracy of quantification.

Despite the advantages of the technique, several issues need to be addressed to fully exploit the potential of metabarcoding in general surveillance programs. The 16S gene and the ITS regions of the rDNA are the most widely used barcodes for inferring phylogenetic relationships among bacteria and fungi because of their high sequence diversity, high number of copies per cell, conserved primer sites and numerous sequences in the databases [57,58]. Since the sequencing of full length of either barcode is not yet feasible with second generation HTS techniques, scientists have focused on sub-regions such as hypervariable regions from V1-V9 in bacteria and ITS1 or ITS2 for fungi [59]. However, these short molecular markers often lack sufficient variability to differentiate closely related taxa and often do not allow the precise species identification of important microorganisms [58,60,61]. This issue is particularly important for plant pathogens because closely related species or strains with very similar or even identical barcode genes may have a completely different pathogenic behavior [59]. This drawback is relevant for fungi, oomycetes and bacteria and it is, therefore, the major limitation of the technique. 

To overcome the problem of specificity, a number of studies have demonstrated the need for more genes to accurately build phylogenetic relationships among complex taxonomical groups. The use of supplementary barcodes in concert with ITS and 16S as the primary barcode has been suggested for the identification of both fungi and bacteria, respectively [60,62]. Similarly, future amplicon metagenomics will likely be based on two or more barcode genes since this would greatly increase the accuracy and details of analyses. Scibetta and co-workers [63] demonstrated that results might significantly change according to primers and ITS regions, highlighting the need for more targets to have a realistic picture of the actual fungal community, which supports an earlier suggestion by Porter and colleagues [64] regarding the use of different markers to study fungal and plant communities. 

Selected barcodes may include both broad range genes (general surveillance) and more specific genes (specific surveillance). For example, the amplification of the elongation factor 1α can efficiently assess the diversity of *Fusarium* spp. [65,66,67], which cannot be differentiated just by using ITS [68]. Other useful target genes include the β-tubulin for the identification of *Penicillium* species [69,70], the calmodulin for *Aspergillus* spp. [61,71], the Ypt1 gene for *Phytophthora* spp. [72,73] and the RPB2 that can be used in conjunction with other markers to reach species identification within many genera [74,75,76]. These loci can also be used alone, without ITS, for specific surveillance projects where the target is a single genus or a restricted number of related taxa [68,77]. 

Regardless of the target barcode gene, the selection of primers is a key aspect that can greatly affect results. A large number of in silico studies have been conducted to identify the best primers for metabarcoding studies [78]. However, a recent investigation of fungal diversity in the phyllosphere using 5 different ITS primer sets, revealed that each set detected around 50% of the overall fungal population, highlighting that most recent metabarcoding studies based on a single primer set did not show a consistent part of the actual microbial diversity [63]. This phenomenon is caused by the fact that microbial genomes can vary in the primer-amplified regions, and primers do not have equal affinity for all possible DNA sequences, consequently inducing a bias during PCR amplification. For example, some taxa may be preferentially or exclusively detected by certain primer sets that vary in one or few bases [73]. Therefore, it is necessary to carefully select primers that are adequate for the desired application, making sure that they allow for the precise detection and identification of the target pathogens. Degenerate primers can increase the detected number of taxa, and also allow for metabarcoding studies using non-standard marker genes [79,80], but the risk of primer slippage can cause a variation in amplicon length, introducing a bias in experiments [81]. Another risk is the potential amplification of host sequences during metabarcoding analyses. This could be particularly relevant when working with bacteria, because the 16S region of plastids and mitochondria is similar to the cyanobacteria one, but the issue can be reduced by selecting appropriate primers [82,83,84]. It is also possible to avoid host DNA coamplification by utilizing blocking oligonucleotides [85,86]. On the other hand, the use of more specific primers targeting a single genus or a restricted number of related taxa may be useful in specific investigations focusing on important taxonomic groups. For instance, *Phytophthora* spp. specific primers targeting the ITS1 region have been used to investigate *Phytophthora* diversity in different environments [87,88,89,90]. Similarly, genus specific primers were utilized to study *Colletotrichum* species associated to olive phyllosphere and carposphere [91] and *Trichoderma* species [92].

An additional source of bias comes from the fact that, in most metabarcoding analysis, similar sequences are clustered together in OTUs (operational taxonomic units) with a 97% or 99% similarity threshold due to the effect of errors introduced during sequencing [93]. This issue can be limited by using algorithms such as Deblur [94], DADA2 [95], and UNOISE2 [96] which correct Illumina-sequenced amplicon errors and allow the user to skip OTU construction, obtaining instead amplicon sequence variants (ASVs). Pauvert and colleagues [97] tested 360 softwares and parameter combinations on mock communities composed of a wide array of Ascomycota and Basidiomycota, identifying DADA2-based approaches as the ones most effective at capturing the composition of the communities. Another way of investigating sub-OTU diversity is through oligotyping, a technique that focuses on specific highly variable sites in sequences to identify diversity in specific target taxa [98]. Oligotyping has been used mainly in studies targeting the 16S region for the analysis of bacterial populations [99,100,101,102], but a recent study demonstrated its reliability also for fungi by identifying *Magnaporthe grisea* and *M. oryzae* at the species level [103]. Attempts to overcome challenges related to the identification of microbial species include the manual analysis of sequences and their use to conduct classical phylogenetic analyses along with validated panels of reference sequences [89,104]. This approach is based on the identification of sequence types (STs), defined as distinct and reproducible sequences [88,89,104]. A subsequent evolution of STs is the definition of the previously mentioned ASVs, defined as individual DNA sequences recovered from a high-throughput marker gene analysis following the removal of spurious sequences generated during PCR amplification and sequencing. The phylogenetic analysis of accurately selected sequences enabled the exploitation of all the available genetic variations within the barcode gene, making it possible to identify taxa with the highest possible level of accuracy. However, it is very time-consuming and requires the existence of well-described and validated reference sequences, which in many cases are not available [59]. Nevertheless, none of the listed bioinformatic methods can overcome the limitations of the selected marker gene and, therefore, the resulting units will not necessarily be phylogenetically and ecologically informative [100].

Another limitation of metabarcoding is that contamination, if present, is increased cycle after cycle, making it particularly relevant when working with low-biomass samples.

### 2.4. Shotgun Metagenomics

Shotgun metagenomics is the untargeted high throughput sequencing of the entire DNA of a sample. It has numerous advantages over metabarcoding, particularly for general surveillance purposes, but analyzing shotgun metagenomics data require several complex operations. After quality control, the reads must be assembled in the contigs of the metagenome, which are then grouped to form the whole genomes of the present microorganisms in a procedure called “binning”. When the microorganisms of interest have low abundance, or when other problems make assembly difficult, it is also possible to perform assembly-free taxonomic profiling, whose efficiency strongly relies upon the availability of reference genomes. Metabolic profiling is then performed, and statistical post-processing analysis is necessary to interpret the newly generated data. All of the cited operations are explained and discussed by Quince et al. [105]. Despite the complexity of the aforementioned steps, we are seeing the release of softwares, such as ATLAS [106], that enable users to perform all the necessary analyses in a single work environment, making the procedure more user friendly.

Shotgun metagenomics has the significant advantage of detecting all microorganisms present in a sample, both fungi and bacteria, without any of the PCR-associated biases that affect metabarcoding. Moreover, the technique has the potential for reaching strain-level resolution, although only for particularly abundant species. Furthermore, shotgun metagenomics can return the entire genome of the detected pathogens, making it ideal for sequencing unculturable ones [107]). However, the technique presents some limitations. When sequencing complex communities, marker genes may only be recovered at low frequency, making taxonomic assignment of all species difficult, impossible or biased toward species whose full genomes are present in databases. Secondly, the sequencing depth required to capture all the community, especially scarcely present species, is much higher than in metabarcoding, causing much higher expenses. Binning, while giving a lot of information and potentially being able to characterize new pathogens, is not feasible when there is high biodiversity, many related strains are present, organism distribution is very uneven and/or low coverage yields fragmented assemblies [93], making it fundamental to use software such as BUSCO [108] and CheckM [109] to test the completeness and quality of assemblies.

### 2.5. Sequencing Methods

The first developed HTS method was 454 pyrosequencing [110] and for a while it was the preferred sequencing method for metabarcoding projects. Today, however, and since 454 pyrosequencing has been discontinued, Illumina has become a more popular choice for both metabarcoding and shotgun metagenomics studies. This technology provides the sequencing method with the best bases/cost ratio, with short (150–300 bps) but high-quality (99.9% accuracy) paired end (PE) sequences. A very high level of accuracy is extremely important in plant pathology because different pathogens are frequently differentiated by few key mutations within the barcode gene. However, successful identification at the species level of an important pathogen such as *Heterobasidium annosum sensu stricto* was also achieved by IonTorrent technology, in which bidirectional amplification and greater read length (400–450 bp) can result in a robust species inference [111].

Illumina and IonTorrent sequencings are two of several second-generation sequencing technologies, all relying on the amplification of base DNA and the production of small reads. A number of third generation sequencing strategies, producing long reads by sequencing directly the DNA molecules, are also available. The most popular is PacBio sequencing, obtaining reads of 30–100 kb. In shotgun metagenomics studies, this sequencing technique provides the user with better average contig length and a higher number of large contigs, resulting in significant enhancements in binning and genome reconstruction [112]. On the other hand, in a metabarcoding study, it allows for sequencing of longer PCR fragments such as the full ITS1-5.8S-ITS2, greatly improving taxonomic identification at the species and phylum levels in general surveillance projects. This type of sequencing was used to study the microbial populations of rust lesions on coffee [113] and to perform community analyses of arbuscular mycorrhizal fungi on soil and root samples [114]. However, PacBio does not perform well with low-quality material and is characterized by higher rates of sequencing errors, which may greatly complicate the identification of known taxa as well as the recognition of putative new ones, still unknown to the scientific community. For this reason, as well as for the higher cost, we are still far from seeing a widespread use of third-generation sequencing in metabarcoding-based plant pathogen surveillance.

Another third-generation sequencing strategy has been developed by Oxford Nanopore Technologies. Nanopore sequencing requires more DNA and remains more susceptible to inhibitors of library construction or sequencing compared to Illumina sequencing, while also having less accuracy, reaching a consensus of around 95% [115]. However, it is faster than Illumina or PacBio, enabling users to detect pathogens within minutes of the start of the sequencing [116]. Consequently, if its accuracy improves, it may become an interesting alternative for screening for quarantine pathogens at customs, an application that requires rapid testing. Due to its small size and the ability to be operated from a simple laptop, the MinION sequencer is also the only current alternative for overcoming situations where physical or legal restrictions prevent the sample transport [117].

### 2.6. Databases

Regardless of the chosen method, the taxonomic attribution of each sequence depends upon alignment to a database. Many tools are available to do this, but the choice of the database is fundamental. The most common databases used in metabarcoding, such as UNITE, SILVA, Greengenes and UniEuk [118,119,120,121], provide users with databases and reference datasets populated with filtered and third-party annotated sequences. The most comprehensive databases for shotgun metagenomics are Genbank and Uniprot, which are not revised and not always reliable [122], therefore users must ensure the accuracy of sequences on the databases regarding the target pathogens. The utilization of curated databases, such as RefSeq [123] and Swissprot [124], is preferable, but they may not contain the sequences of many organisms of potential interest, given the low number of whole genome sequencing (WGS) projects conducted on plant pathogens.

When the scope of the surveillance is limited, such as in specific surveillance studies, the building of in-house databases for the target species [125], although time-consuming, is a valuable option to be considered.

## 3. Application of HTS in Plant Disease Diagnosis

### 3.1. Surveillance

**Figure 1:**
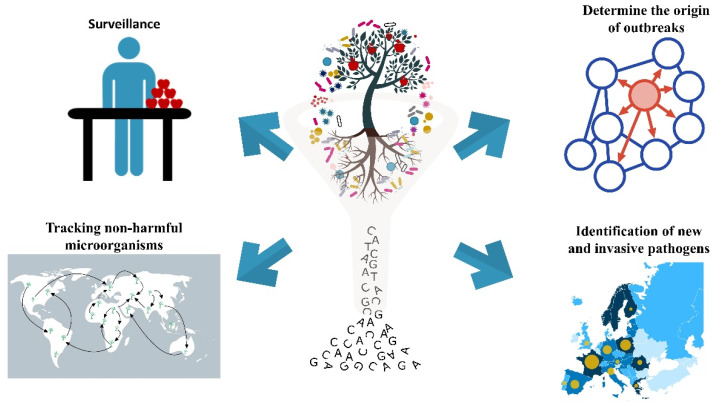
Possible applications of shotgun and amplicon-based metagenomics.

Shotgun and amplicon-based metagenomics have a wide array of possible applications (Figure 1, Table 1). Regarding surveillance, these methods are well suited for the detection of soilborne pathogens, including the genera *Rhizoctonia* spp., *Fusarium* spp., *Verticillium* spp., *Sclerotinia* spp., *Pythium* spp., and *Phytophthora* spp., which affect many different crops [126,127,128,129]. These pathogens are notable because of the severe yield losses they cause which can reach up to 75%, and because they can form fruiting bodies such as microsclerotia, sclerotia, chlamydospores, or oospores that are able to survive in the soil for years, without the presence of the host [130]. Symptoms induced in host plants by these pathogens are often similar, and include root rot, root blackening, wilt, yellowing, stunting or seedling damping-off, bark cracking and twig or branch dieback [131]. These characteristics make soilborne diseases difficult to diagnose and control using traditional identification methods. The use of both amplicon-based and shotgun metagenomics can address some of the problems, allowing the detection of pathogens in the absence of a host and in an untargeted manner that avoids assumptions and/or biases.

The detection of seedborne pathogens, which tend to become widely distributed across many nations due to current trends in specialized production areas [12,13,14], may also benefit from use of metagenomics. Untargeted detection methods provide the ability to quickly test for the presence of multiple pathogens in a single analysis. For example, Franić and colleagues [18] used ITS2 sequencing to screen fungal pathogens in 58 traded seed lots of 11 gymnosperm and angiosperm tree species from North America, Europe, and Asia. Using ASVs instead of OTUs, the authors were able to detect contamination of seeds with the fungal pathogen, *Diaporthe alleghaniensis,* in seed lots of *Larix gmelinii* from Asia, and *Microascus cirrosus* in seed lots of *Picea abies* from Europe. Furthermore, the use of HTS would also improve the detection and identification of airborne pathogens. Although there are several ways to monitor fungal spores in the atmosphere [132], one of the most common approaches is the use of Hirst traps [133], which is based on the deposition of spores on sticky tapes on rotating drums. The analysis of the spores present on the sticky tapes is traditionally done by microscopic observation, which is a time-consuming and laborious task requiring considerable expertise in spore identification [134]. Results obtained by this method can be unreliable due to varying levels of expertise of the person performing the analysis, viewing of only portions of the tape rather than the complete tape, and the presence of plant material, insects, dust, and other particles that complicate the analysis [38,135]. Moreover, bacterial and fungal spores can travel over long distances through the air, often arriving in areas where they are not expected, without routinely executed targeted molecular assays, such as qPCR and LAMP [15,16,31]. Metabarcoding and shotgun metagenomics are untargeted methods that are not subject to the previously described issues. Therefore, the use of spore traps together with metagenomics can improve the ability to detect the presence of a wide array of pathogens and microorganisms in general, enabling authorities to provide more informed decisions on appropriate crop protection strategies. Tremblay and colleagues [111] utilized IonTorrent technology to sequence the ITS1 in 390 aerial and insect trap samples from several Canadian locations at high risk of fungal spread, and were able to identify pathogen species such as *Heterobasidium annosum sensu stricto* and *Heterobasidium abietinum/parviporum*. These species are endemic in Europe and Asia [136,137] but had also been reported in the United States [138]. Their identification in the Canadian samples enabled them to raise a warning against a potential threat of invasion of these pathogens into Canada. The atmospheric mycobiota was also studied using these techniques to investigate the interactions between plants and the airborne fungal communities [26,103]. Franco Ortega and colleagues [103], analyzed the airborne fungal mycobiome in a rice field, collecting spores on a daily basis from spore traps throughout the summer. Using oligotyping, they identified important pathogens such as *Magnaporthe oryzae* and *Magnaporthe grisea* at the species level and determined the phenological phase of rice when the pathogens were most abundant, correlating their abundance with temperature and precipitation. Whereas, Abdelfattah and colleagues [26], investigated the influence of aerial microbiome on the composition of the mycobiome associated with leaves, flowers, and fruit of table grapes. Surprisingly, the authors found that the air surrounding the grape phyllosphere has a significantly higher level of fungal diversity than the grape phyllosphere itself. They also found that 92% of the aerial mycobiome originated from the local plants (grapevines). In contrast, only 4–35% of the plant-associated fungi originated from the air, confirming that plants are the major source of their own microbiota, while atmosphere is a complementary source of the plant mycobiome.

Fungal abundance and diversity detected in the cited studies were dependent on the use of spore traps and spore recovery as well as biases introduced by primer selection. Aguayo and colleagues [139] demonstrated that filter paper-based traps are better suited for metabarcoding studies than vaseline (petrolatum) and paraffin wax-based traps, and that shaking-based and rubbing-based spore recovery methods generally produce higher mean diversity than direct grinding of the filter paper spore traps. Further studies are needed, however, to reach a consensus regarding the best way to monitor airborne fungal pathogens using amplicon-based HTS. Nanopore sequencing is currently the most rapid HTS technology and, although problems in its accuracy still need to be addressed, it could become the most appropriate for the monitoring of fungal spores in air samples since a timely diagnosis is particularly important to control diseases caused by pathogens mainly diffused by airborne spores.

### 3.2. Identification of Emerging Pathogens

Shotgun metagenomics, and to a lesser extent amplicon metagenomics, have great potential for identifying emerging pathogens. Amplicon metagenomics can enable to identify new sequences from still unknown microorganisms and provide preliminary information about their phylogenetic collocation [89]. Shotgun sequencing can offer significant amounts of information on a new causal agent of plant diseases, as the near-complete or entire genome of an organism can be obtained by sequencing [107]. This sequence data can greatly contribute to risk assessment by national regulatory agencies. The technique does, however, include a risk of prematurely identifying commensal or saprophytic organisms as pathogens. Subsequent verification of the Koch’s postulates is desirable, but it can be very time-consuming in the case of obligate pathogens, diseases caused by multiple pathogens, or diseases linked to environmental factors [142]. Nevertheless, HTS-based analyses and other molecular approaches (e.g., PCR) can be used, if not to prove causality, at least to quickly demonstrate a strong association between the presence of microorganisms and disease symptoms, as was done for Carrot Yellow Leaf Virus (CYLF) and carrot internal necrosis by Adams and colleagues [140]. Over two decades ago, Fredericks and Relman [143] proposed some guidelines for sequence-based determination of a causal relationship between microorganisms and diseases.

### 3.3. Determine the Origin of Outbreaks

Rapid detection of outbreaks and determination of their origin using the genome sequence of a pathogen is gaining prominence in applications related to food security [144,145], and in both human and veterinary health [146]. DNA sequencing has been used in forensic science for many years [147], and FDA is using HTS and genome sequencing to identify and track specific strains of foodborne pathogens [148]. Despite this demonstrated applicability, the use of shotgun metagenomics to trace the origin of plant pathogen outbreaks has not been widely explored, although such studies are beginning to be reported. In this regard, Hubbard and colleagues [141] used genomic sequencing to determine that the UK population of *Puccinia striiformis* f. sp. *tritici*, pathogen of wheat, comprises 4 lineages and that these were derived from exotic isolates that displaced previous strains present in native populations. Their study also demonstrated the utility of using a metatranscriptomic approach to study obligate parasites in plants like wheat, whose large genome (17 Gb) relative to the pathogen (110 Mb) would make a shotgun metagenomic approach problematic. Another approach to address the problem of a high level of non-target, host sequences is the use of fungal-sequence hybridization capture, that potentially combines high sensitivity and specificity. This technique has been proposed for use in the survey of human pathogens [149] but has not been applied to the analysis of plant pathogens.

### 3.4. Tracking Non-Harmful Microorganisms

HTS-based analyses produce taxonomic data that are not directly relevant to pathogen diagnosis, since HTS will detect most of the microbial taxa present in a sample if conducted properly. These data are far from useless. As previously stated, microorganisms can be non-harmful in their natural environment but become pathogens when they colonize a new niche [24]. Tracking the distribution of all microbial taxa, not just the pathogens, can provide information if a new disease subsequently appears. This is especially true if the survey is undertaken using shotgun metagenomics, as it would potentially contain genome sequence data on the new pathogen, already known. Data produced by comprehensive HTS-based surveys allow researchers to compare the genome of organisms before and after the “lifestyle switch” from non-pathogenic to pathogenic one. Increased knowledge of this process is essential for the development of efficient and biologically-based plant disease control measures [150].

### 3.5. Limitations

Despite the advantages and opportunities of HTS-based diagnostics presented in this review, it still has limitations that should be taken into consideration in the interpretation of results. First, detection of a pathogen can be due to the presence of DNA from dead cells that are no more able to cause disease, thus providing a false positive result. This problem can be potentially overcome by isolation and culturing of the pathogen from the sample to demonstrate its viability [18]. However, this need dramatically increases the time needed to conduct an analysis and it can only be done with culturable microorganisms. Other methods to differentiate living and dead cells are the use of propidium-monoazide (PMA) and RNA-based analyses [151], but both methods greatly complicate analyses.

A second important limitation is the risk of erroneously identifying pathogens based on conserved sequences that actually originate from non-pathogenic organisms whose sequences are not available in public databases [152]. This concern is particularly relevant for shotgun metagenomics analyses, since the number of sequenced microbial genomes is still rather small.

Finally, it is particularly important when using HTS-based methods for general surveillance to have a comprehensive knowledge of the naturally-occurring microbial communities present at location being evaluated, as HTS has the potential to identify new pathogens species that were actually already present in the location, but unreported. This type of misidentification could cause authorities to put in place costly quarantine measures to control microorganisms that were always present at a certain location and that do not pose new threats. To mitigate this risk, Belgium and other countries have already started national HTS-based surveys to detect viruses present in cultivated and wild plants within their territories [153].

Finally, the application of the discussed techniques is limited by the expertise required to properly manage HTS-generated data. Even if many softwares, such as QIIME and Mothur [154,155], can be used remotely and with a user friendly interface through the Galaxy web platform [156], an in-depth knowledge of the commands and operations necessary to perform the analyses remains necessary to choose the best methods and avoid mistakes. Being conscious of the risks is of paramount importance when establishing HTS-based diagnostic protocols, as the detection of quarantine pathogens can have significant economic consequences, both for exporting and importing countries [157]. Examples of this can be seen in the testing against *Phytopythium helicoides* and *Phytopythium vexans* for the import of *Actinidia* plants in New Zealand [154], or in the additional measures taken by South Africa to ensure that citrus exports to EU are not contaminated by *Phyllosticta citricarpa* [155].

## 4. Conclusions and Future Studies

High-throughput sequencing of the microbiome has great potential for diagnostic purposes. It is particularly promising for the surveillance of soilborne, seedborne, and airborne pathogens, but also for the identification of new pathogens and for determining the origin of outbreaks. Metabarcoding, mainly due to its low cost, is currently the technique with the greatest number of potential applications in monitoring the presence of pathogens, and is particularly valuable for use in specific surveillance projects where the target is a single genus of fungi or bacteria. Shotgun metagenomics, while having important advantages over metabarcoding, is more expensive and is most appropriate under specific conditions, such as low biodiversity, low number of related strains, and relatively even organism abundance. Even though both metabarcoding and shotgun metagenomics approaches still suffer from low precision, this issue can be limited by carefully choosing primers and bioinformatic algorithms. Illumina remains the most popular sequencing technique for all kinds of HTS-based pathogen detection, but the use of third generation sequencing could spread in the future due to its higher read length. In particular, the speed of nanopore sequencing would offer an interesting alternative for the detection of airborne pathogens, whose timely control greatly benefits from early detection. As the field of metagenomic analyses of the microbial world is rapidly evolving, it is expected that additional technological innovations and advances in bioinformatics will greatly accelerate the use of metagenomics to address critical problems related to plant pathology and the global exchange of plant materials and harvested crops.

## Figures and Tables

**Table 1:** Representative examples of studies focusing on the use of high-throughput sequencing (HTS) methods to detect plant pathogens.

Field of Application	Application Scope	Techniques Used	Main Detected Pathogens	Ref.
Traded seed lots of gymnosperm and angiosperm tree species	General surveillance	Illumina sequencing, metabarcoding, ASV (amplicon sequence variants) assembly	Several seedborne pathogens	[18]
Aerial samples in rice field.	General surveillance	Illumina sequencing, metabarcoding, olygotyping, OTU (operational taxonomic unit) assembly.	*Magnaporthe oryzae, Magnaporthe grisea*	[103]
Leaves, flowers and fruits of olive	General surveillance	454 Pyrosequencing, Metabarcoding, OTU assembly, classical phylogenetic analysis of sequences.	*Collethotricum* spp., *Pseudocercospora cladosporioides*	[104]
Aerial and insect trap samples, soil samples	General surveillance	Metabarcoding, IonTorrent sequencing, OTU assembly.	*Heterobasidium annosum sensu stricto*, *Heterobasidium abietinum/parviporum*, *Phytophthora* spp.	[111]
Field samples of barley, durum and soft wheat	Specific surveillance	Illumina sequencing, metabarcoding, Genus-specific primers, OTU assembly.	*Fusarium* spp.	[68]
Soil and root samples of ornamental potted plants	Specific surveillance	454 Pyrosequencing, metabarcoding, genus-specific primers, classical phylogenetic analysis of sequences.	*Phytophthora* spp.	[89]
Soil and water samples from forests and plantations	Specific surveillance	454 Pyrosequencing, metabarcoding, genus-specific primers, classical phylogenetic analysis of sequences.	*Phytophthora* spp.	[126]
Infected citrus infected psyllid	Pathogen identification	454 Pyrosequencing, shotgun metagenomics, complete pathogen genome sequencing.	*Candidatus Liberibacter asiaticus*	[107]
Carrot samples	Identification of emerging pathogens	Illumina sequencing, shotgun metagenomics.	Carrot Yellow Leaf Virus	[140]
Infected wheat leaves	Determining outbreak origin	Illumina sequencing, shotgun metatranscriptomics.	*Puccinia striiformis* f. sp. *Tritici*	[141]
